# Characterization of a *de novo* assembled transcriptome of the Common Blackbird (*Turdus merula*)

**DOI:** 10.7717/peerj.4045

**Published:** 2017-12-13

**Authors:** Sven Koglin, Daronja Trense, Michael Wink, Hedwig Sauer-Gürth, Dieter Thomas Tietze

**Affiliations:** 1 Institute for Pharmacy and Molecular Biotechnology, Ruprecht-Karls-Universität Heidelberg, Heidelberg, Germany; 2 Zoological Institute and Museum, University of Greifswald, Greifswald, Germany; 3 Current affiliation: Natural History Museum Basel, Basel, Switzerland

**Keywords:** RNAseq, Transcriptomics, SNP, Common blackbird, Ornithology

## Abstract

**Background:**

In recent years, next generation high throughput sequencing technologies have proven to be useful tools for investigations concerning the genomics or transcriptomics also of non-model species. Consequently, ornithologists have adopted these technologies and the respective bioinformatics tools to survey the genomes and transcriptomes of a few avian non-model species. The Common Blackbird is one of the most common bird species living in European cities, which has successfully colonized urban areas and for which no reference genome or transcriptome is publicly available. However, to target questions like genome wide gene expression analysis, a reference genome or transcriptome is needed.

**Methods:**

Therefore, in this study two Common Blackbirds were sacrificed, their mRNA was isolated and analyzed by RNA-Seq to *de novo* assemble a transcriptome and characterize it. Illumina reads (125 bp paired-end) and a Velvet/Oases pipeline led to 162,158 transcripts. For the annotation (using Blast+), an unfiltered protein database was used. SNPs were identified using SAMtools and BCFtools. Furthermore, mRNA from three single tissues (brain, heart and liver) of the same two Common Blackbirds were sequenced by Illumina (75 bp single-end reads). The draft transcriptome and the three single tissues were compared by their BLAST hits with the package VennDiagram in R.

**Results:**

Following the annotation against protein databases, we found evidence for 15,580 genes in the transcriptome (all well characterized hits after annotation). On 18% of the assembled transcripts, 144,742 SNPs were identified which are, consequently, 0.09% of all nucleotides in the assembled transcriptome. In the transcriptome and in the single tissues (brain, heart and liver), 10,182 shared genes were found.

**Discussion:**

Using a next-generation technology and bioinformatics tools, we made a first step towards the genomic investigation of the Common Blackbird. The *de novo* assembled transcriptome is usable for downstream analyses such as differential gene expression analysis and SNP identification. This study shows the importance of the approach to sequence single tissues to understand functions of tissues, proteins and the phenotype.

## Introduction

High-throughput or next-generation sequencing (NGS) is a useful tool not only for organisms with established reference genomes and transcriptomes, but also for creating reference transcriptomes and genomes for non-model organisms. Consequently, with the still ongoing development of NGS technologies and with the help of bioinformatics tools for the analysis of the resulting large data sets, numerous publications concerning avian transcriptome or genome characterizations have already been published (Lerner & Fleischer, 2010; Basch et al., 2011; Botero-Castro et al., 2013; Shanks et al., 2013; Kraus & Wink, 2015; Xu et al., 2016).

NGS technologies have been adopted by a wide range of scientific disciplines, e.g., diagnostics/medical research (Shanks et al., 2013), developmental biology (Basch et al., 2011), evolutionary research (Botero-Castro et al., 2013), ecotoxicology (Xu et al., 2016). Additionally, in recent years, ornithologists started to make use of these technologies. Especially in the fields of (avian) phylogenomics and comparative genomics, NGS technologies have become a commonly used tool. Furthermore, as in other disciplines, NGS technologies (especially RNA-Seq) have a great potential to understand phenotypes and adaptation to different ecological conditions (Lerner & Fleischer, 2010; Kraus & Wink, 2015).

Most studies dealing with avian transcriptomics or genomics use the available sequenced and (more or less) annotated genomes of chicken (*Gallus gallus* f. *domestica*) provided by Hillier et al. (2004) and Zebra Finch (*Taeniopygia guttata*) provided by Warren et al. (2010). However, it is also feasible to establish *de novo* transcriptomes of birds without having known reference genomes or transcriptomes. For example, Künstner et al. (2010) conducted a comparative genomics approach by sequencing ten avian non-model transcriptomes. Feldmeyer et al. (2011) were the first to assemble a non-model species transcriptome from Illumina short reads by comparing assembler performances and Ekblom et al. (2014) characterized the transcriptome from three tissues of the House Sparrow (*Passer domesticus*) and used this data to investigate typical genes associated with immune functions. Transcriptome and genome of the Blue Tit (*Cyanistes caeruleus*) were described (Mueller et al., 2016) to identify polymorphisms and candidate genes for sex determination and a transcriptome of the House Finch (*Haemorhous mexicanus*) spleen was characterized by Zhang et al. (2014a) understanding the evolution in avian spleen, genetically. Furthermore, Qu et al. (2013) sequenced and assembled the genome of the Ground Tit (*Pseudopodoces humilis*) to survey avian adaptation to high altitudes and, recently, Laine et al. (2016) used an NGS approach to provide a Great Tit (*Parus major*) genome and investigated the selection for cognition. Another genome of a species, which is closer related to the Blackbird than the Great Tit, is the *Ficedula* flycatchers’ genome with a size of 1.1 Gb (Ellegren et al., 2012). A comparison of 48 bird genomes was drawn by Zhang et al. (2014b) and they detected millions of highly constrained elements. Videvall et al. (2015) and Vijayakumar et al. (2015) investigated the changes of gene expression as a response of birds to infection and Chung et al. (2015) surveyed the adaptation in avian gastric and immune defense system.

Furthermore, transcriptomes offer the opportunity to directly investigate the protein coding regions of genes, to analyze gene expression patterns in different tissues and developmental stages or differences as well as to identify single nucleotide polymorphisms (SNP) (Vera et al., 2008; Elmer et al., 2010; Renaut, Nolte & Bernatchez, 2010; Wolf et al., 2010; Cahais et al., 2012).

One of those birds with, so far, no published reference genome or transcriptome is the Common Blackbird (*Turdus merula*). The Blackbird is a Eurasian woodland species, but became one of the most common bird species found in urban areas in Europe. Its successful colonization of European cities started 200 years ago. Several studies have been conducted using the Blackbird as an experimental organism to investigate behavior (e.g., vocalization) (Dabelsteen & Pedersen, 1990; Partecke, Van’t Hof & Gwinner, 2004) and physiology (Cresswell, 1998; Partecke, Schwabl & Gwinner, 2006) in relation to adaptations to the urban environment. DNA studies have been performed to identify differences between urban and rural Blackbirds. For example, Partecke, Gwinner & Bensch (2006) used genetic markers, but found that urban and rural populations were very similar. The Blackbird was genetically analyzed using eight microsatellites as molecular marker (Segelbacher et al., 2008) as well as two mitochondrial and one nuclear genes (Rodrigues et al., 2016). A mitochondrial genome of the Blackbird was published by Peng, Yang & Lu (2015) with a total length of 16,730 bp and they found 13 protein coding genes. The mitochondrial genome of the congeneric Rufous-bellied Thrush (*Turdus rufiventris*) was described, containing 16,669 bp and they detect 13 protein coding genes, too (Gomes de Sá et al., 2015). Franchini et al. (2017) analyzed blood of twelve Blackbirds using high-throughput transcriptomics and detected few differently expressed genes in individuals of different migratory behavior.

Further transcriptome and genome analyses will help to link the previously observed phenotypic phenomena of the Blackbird with molecular data. This would enable ecologists and evolutionary biologists to investigate the urbanization of the Blackbird with a wider range of molecular methods and their migration strategies. This study has been conducted to establish and characterize a first *de novo* assembled Common Blackbird overall transcriptome. For this purpose, we caught two male Blackbirds from an urban and a rural population, sequenced the mRNA from 14 types of tissue by using Illumina technology and used bioinformatic tools for data processing and the assembly and annotation of a transcriptome.

## Materials & Methods

### Collection of the birds

For this study, two adult male Common Blackbirds (*Turdus merula*) were collected during the breeding season (March to July). Individuals were caught in the forest of Eberbach (rural habitat) and in the Botanical Garden of Heidelberg University (urban habitat). Collected birds were killed by decapitation and 14 types of tissue (brain, kidney, liver, spleen, heart, small intestine, large intestine, stomach, blood, lung, testicle, pectoral muscle, retina, skin) were removed under sterile conditions. The extracted tissues were immediately stored in liquid nitrogen. Birds were sacrificed in accordance to the German Animal Welfare Act and with permission of the Regierungspräsidium Karlsruhe (Aktenzeichen: 55–8853.15/Uni HD). The city of Eberbach allowed work in their forest (Aktenzeichen: 54.01–856.8603.07:0001).

### RNA extraction, cDNA synthesis, and Illumina sequencing

Before extracting the RNA, the removed tissues were initially homogenized using a mortar and further homogenized using a swing mill (MM400; Retsch GmbH, Haan, Germany). During homogenization, the samples were kept on liquid nitrogen. The RNA was extracted from pre-pools (pool 1: brain, kidney, liver, spleen; pool 2: heart, small intestine, large intestine, stomach; pool 3: blood, lung, testicle; pool 4: pectoral muscles, retina, skin) of the removed 14 different tissues and three single tissues (brain, heart, liver) of each individual using the Universal RNA Purification Kit (Roboklon, Berlin, Germany). The extraction was conducted according to the manufacturer’s protocol, but additional washing steps were performed. The RNA extracted from the pre-pools was pooled again per individual using a volume that contained 6 µg of RNA from each pre-pool. After that, quality and quantity of the pre-pools were checked using a 1.4% agarose gel and concentrations (in ng/µL) were measured spectrophotometrically (wavelengths: 230 nm, 260 nm, 280 nm; Biochrom Biowave II; Biochrom Ltd., Cambridge, UK). Additionally, the extracts were checked for DNA contamination by performing PCR with mitochondrial marker genes. To retrieve higher RNA qualities, the RNase-free DNase set (Qiagen, Hilden, Germany) was used additionally. This step was conducted according to the manufacturer’s protocol, but additional washing steps were performed. The quality and quantity of the samples was checked again as described before. The extracted RNA was stored at −80 °C. Initial RNA Quality control for the single tissues was performed on the Agilent BioAnalyzer (Agilent Technologies, California, USA) using the RNA 6000 Nano kit (Agilent Technologies) and concentration was determined by the Invitrogen Qubit (Thermo Fisher Scientific, Waltham, MA, USA) using the RNA BR reagents (Thermo Fisher Scientific).

All library preparations for Illumina sequencing were conducted in the CellNetWorks Deep Sequencing Core Facility of Heidelberg University by David Ibberson. For the isolation of the mRNA from the whole RNA samples, the NEBNext Poly(A) mRNA Magnetic Isolation Module (New England BioLabs Inc., Ipswich, MA, USA) was used. Furthermore, the NEBNext Ultra Directional RNA Library Prep Kit for Illumina (New England BioLabs Inc.) was employed with the NEBNext Multiplex Oligos for Illumina (New England BioLabs Inc.) for cDNA synthesis and addition of barcode sequences.

The sequencing of the pre-pools was performed using the HiSeq 2000 with v3 chemistry from Illumina (San Diego, CA, USA) and 125 bp paired-end (PE) reads. Singletons were not kept for further analysis. The sequencing of the three single tissues was performed using the NextSeq 500 (Illumina, San Diego, CA, USA) with the NextSeq 500/550 High Output Kit v2 (75 cycles; Illumina). Here, sequencing with 75 bp single-end (SE) reads produced approximately 30 million reads per sample.

### Preprocessing of the raw sequences

The PE sequencing of the pools led to two read files per sample (forward and reverse). These two files per sample and the resulting sequences of the three single tissues were quality checked using FastQC 0.11.2 (Andrews, 2014). Adaptors were trimmed using Trimmomatic 0.33 (Bolger, Lohse & Usadel, 2014) and FastQC was used again to check the trimming results. Additionally, nucleotides from the beginning and the end of a sequence were trimmed, if their quality score was below the standard value of 3. The minimal read length was kept as 36.

### *De novo* assembly and annotation of the transcriptome and alignment of the reads

For the *de novo* assembly of the transcriptome and the single tissues the assembling tools Velvet 1.2.10 (Zerbino & Birney, 2008) and Oases 0.2.08 (Schulz et al., 2012) were used. Before starting the assembly, the two PE files of each individual had to be shuffled using the shuffleSequences_fastq.pl function of Velvet. After that, the *de novo* transcriptome assembly and the assembly of the three single tissues were conducted using the reads of both individuals. In Velvet, the k-mer length was set to 61 (chosen following empirical testing with values for k-mer from 37 to 87), the minimal contig length was set to 100 and the expected coverage was set to one. In Oases, the minimal transcript length was set to 200. To reduce the redundancy of the *de novo* assembled transcriptome the cd-hit-est function of the software CD-HIT 4.6 (Li & Godzik, 2006) was used with default options.

To functionally annotate the *de novo* assembled transcriptome, the stand-alone software tool BLAST+ 2.3.0 (Camacho et al., 2009) was used as described by De Wit et al. (2012) to retrieve additional functional information about the annotated transcripts. For this, also the scripts BlastParse.pl and totalannotation.py provided by De Wit et al. (2012) were used. The transcriptome was annotated against the complete non-redundant, Swiss-Prot, and TrEMBL protein databases (downloaded 2016-02-29), summarized in “unfiltered database”. The blastx function was used to annotate nucleotide sequences against the protein database. The threshold e-value was set to 10^−6^ and the required word size for a match was set to three. Furthermore, the BLOSUM62 matrix was used. Only the matches that were meeting the above mentioned criteria and that had the highest hit identity for the respective transcript (as defined by BLAST+) were considered for further analyses.

For the alignment of the reads to the *de novo* assembled transcriptome, an index of the transcriptome was built using the bowtie2_build function of Bowtie2 2.2.6 (Langmead & Salzberg, 2012), the software that was used for the whole alignment, setting only the no-unaligned option. The resulting SAM files were converted to BAM files using SAMtools 1.2 (Li et al., 2009).

Before identifying SNPs, the aligned reads were sorted by using the sort function of SAMtools. Afterwards the SNPs were identified using the mpileup function of SAMtools, skipping the indels and redoing base-alignment quality assessment. The output was transferred to the call function of BCFtools 1.3.1 (Li et al., 2009), skipping indels, calling multiallelic and rare-variant SNPs, dropping invariable sites, and using 10^−6^ as prior float expected substitution rate. Following the identification, SNPs were filtered for their quality score using the filter function of BCFtools and a threshold of 60. The transition to transversion ratio was calculated with the stats function of BCFtools.

### Data analysis

NGS data were processed on bwForCluster MLS&WISO (http://www.bwhpc-c5.de) and HUSAR (http://www.dkfz.de/gpcf/hs_home.html). If not stated differently, all figures were produced using R (R Core Team, 2015) and the package VennDiagram (Chen & Boutros, 2011) in R.

**Figure 1:**
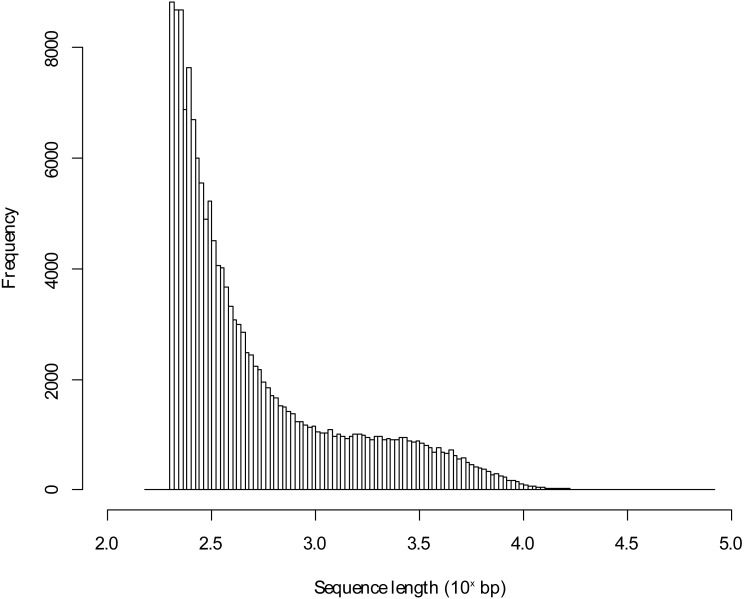
Length distribution of assembled transcriptome sequences. The sequence lengths in bp were log_10_ transformed.

## Results

The sequencing of the pooled Blackbird mRNA led to 48,898,319 PE reads for the urban individual and to 68,085,209 PE reads for the rural Blackbird. Following preprocessing, the mean sequence length was 122.11 bp and 122.04 bp, respectively. The *de novo* transcriptome assembly led to 162,158 transcripts using the reads of both individuals and has a length of 156,342,963 bp. The mean transcript length in this *de novo* assembled transcriptome was 964.14 bp with a maximum transcript length of 81,373 bp (annotated to human titin). The sequence length distribution for the *de novo* assembled transcriptome is shown in Fig. 1. Using BLAST+ with the unfiltered database, 48,607 (29.98%) of all contigs could be annotated to entries in the protein database, but 11.88% of those contigs were annotated to proteins characterized as unknown, predicted, putative or hypothetical in their description so that there was no functional information about those hits (in the following referred to as poorly characterized hits). Table 1 presents the number of contigs that could be annotated according to the percentage of hit identity (as defined by Blast+). It shows that the unfiltered approach led to a high percentage of hits. In some cases, several *de novo* assembled contigs matched the same target in the database.

**Figure 2:**
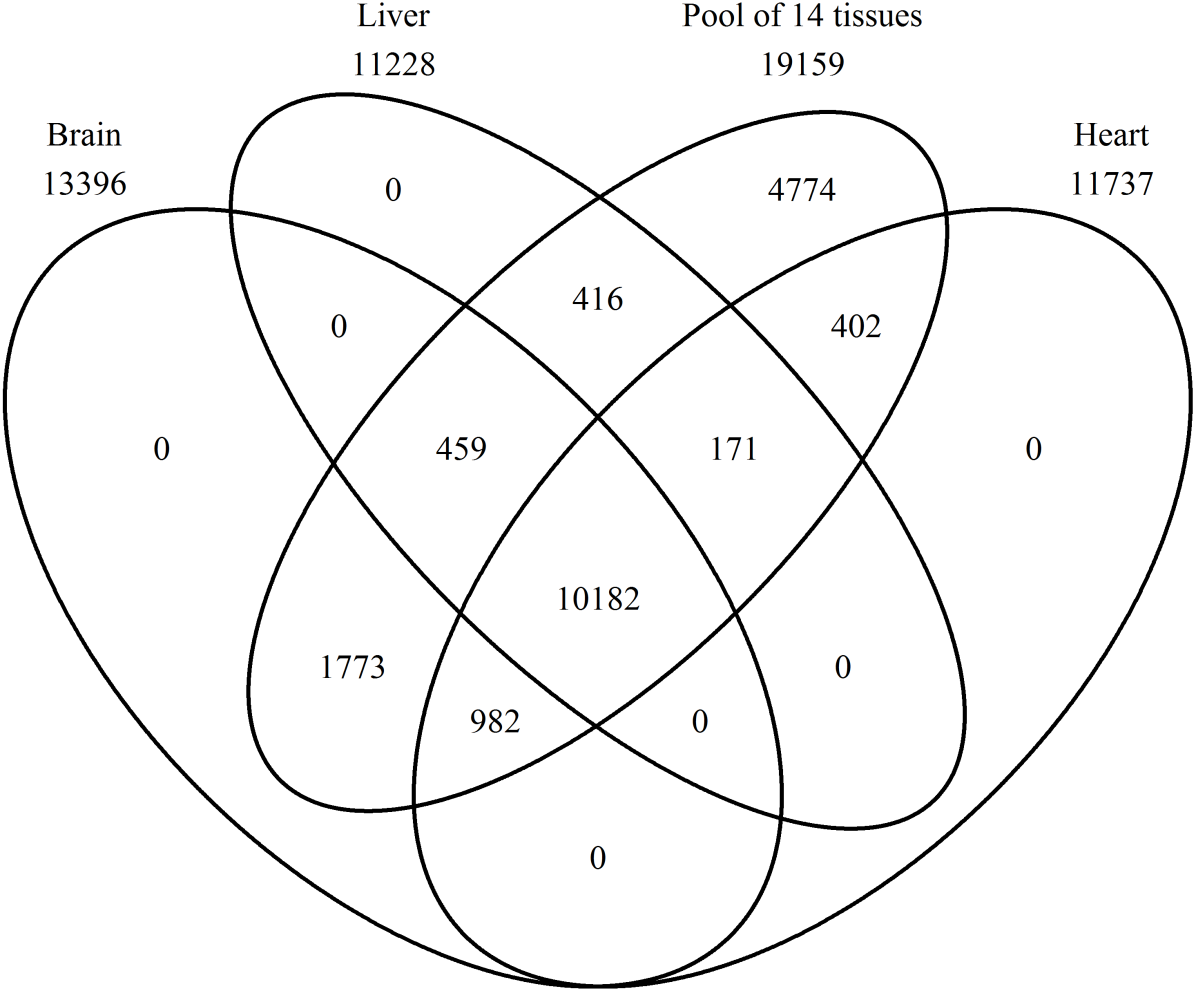
Number of expressed genes. Venn diagram representing the number of expressed genes shared by the three single tissues and the mRNA pool.

A Venn diagram was constructed using protein names for a comparison of the proteins found in the brain, heart, liver, and the pool of 14 tissues (Fig. 2). In the pool of 14 tissues, 19,159 BLAST hits were found, in the brain 13,396, in the liver 11,228, and in the heart 11,737 (Tables S1–S4 with a k-mer length of 61 and cd-hit standard). Brain, heart, liver, and the pool shared 10,182 BLAST hits, and no single BLAST hit occurs in either brain, liver or heart alone, indicating that the PE pool RNA-Seq approach covered all genes transcribed in the individually studied tissues. The 4,774 BLAST hits unique to the pool, are thus from the eleven remaining tissue samples.

**Table 1:** Annotation results of the unfiltered protein database according to the percentage of identity match and after filtering out BLAST hits with unknown, predicted and hypothetical proteins (only well characterized hits). The percentages refer to the total number of transcripts in the assembled transcriptome (162,158). Each identity match category contains the hits that had at least the stated identity match (e.g., 50% match category: all hits had an identity match of at least 50%).

		All	Good hits	50% match	60% match	70% match	80% match	90% match	95% match	100% match
Unfiltered db	Total	48,607	42,833	37,372	31,613	24,595	16,891	8,780	4,512	702
	%	29.98	26.41	23.05	19.50	15.17	10.42	5.41	2.78	0.43

The most expressed gene unique to the brain is synaptosomal-associated protein 25 with 4.6%, to the heart myosin regulatory light chain 2A, cardiac muscle isoform with 21.8%, to the liver fibrinogen alpha chain with 9.5%, and to the pool non-described proteins with 8.2%, but for the pool it should be considered that all proteins with no description are included in the 8.2% (Table 2).

**Table 2:** The five most expressed genes, which occur only in brain, heart, and liver, respectively, as well as in the pool, ordered by decreasing percentage. Percentages were calculated by the mean counts (all doubles were excluded previously) of the two Blackbirds and the sum of these means; for the single tissues, only unique genes were considered.

Tissue	BLAST hit	Percentage
Brain	Synaptosomal-associated protein 25	4.6%
	Neuronal membrane glycoprotein M6-a	3.1%
	Protein AF1q	2.3%
	Glutamate receptor ionotropic, NMDA 1	2.2%
	Neuron-specific protein family member 2	1.9%
Heart	Myosin regulatory light chain 2A, cardiac muscle isoform	21.8%
	Myosin light chain 1, cardiac muscle	18.3%
	Troponin T, cardiac muscle isoforms	13.7%
	Titin	7.8%
	Creatine kinase S-type, mitochondrial	6.4%
Liver	Fibrinogen alpha chain	9.5%
	Kininogen-1	8.5%
	Deleted in malignant brain tumors 1 protein	5.8%
	Phenylalanine-4-hydroxylase	5.7%
	Uncharacterized protein {ECO:0000313 |Ensembl:ENSOANP00000025219}	3.8%
Pool	No description	8.2%
	Uncharacterized protein {ECO:0000313 |EMBL:KNC36522.1}	1.4%
	Serum albumin	0.8%
	Ovoinhibitor	0.8%
	Serine/threonine-protein kinase pim-1	0.8%

Using SAMtools and BCFtools, 144,742 SNPs were identified on 29,257 individual contigs. Consequently, 0.09% of the nucleotides contained in the *de novo* assembled transcriptome were SNPs and 18% of the assembled contigs carried SNPs. The transition to transversion ratio in the transcriptome is 2.27. Further information on the SNPs is provided in Table S5.

## Discussion

In this study, tissue samples of two male adult Common Blackbirds from an urban and a rural habitat were used to *de novo* assemble a reference transcriptome for this bird species. Although equal amounts of input material were used (cDNA), sequencing of the mRNA of two Blackbird individuals led to different amounts of reads for each individual. This can be ascribed to possible differences that could have been introduced during sampling and sample preparation as well as to technical artifacts during the sequencing procedure rather than it could be explained by the biological variation between individuals. This has been stated already by Ekblom et al. (2014) to describe the difference between the overall sequencing reads although equal amounts of cDNA have been used. Considering the mean and maximum length of the *de novo* assembled transcriptome as well as the sequence length distribution of the transcriptome, we assume that this transcriptome possibly covers a wide range of post-processed mRNAs, given that the length of those polynucleotides can vary from tens to tens of thousands of nucleotides.

To obtain a high degree of information for the transcriptome assembly, a relatively high number of PE short reads was used during Illumina sequencing. In comparison with the *de novo* assembled transcriptome of the House Sparrow (15,250 assembled transcripts) conducted by Ekblom et al. (2014), the Blackbird assembly led to a more than ten times higher number of transcripts (162,158 assembled transcripts). Such a discrepancy can occur due to the differences in the used sequencing technology and in the computational processing of the reads. But the assembled blood transcriptome of the Blackbird yielded in 118,813 likely coding sequences, which is close to our resulting assembled transcripts for 14 tissues. In sum, more than 100 million PE reads were used for our Blackbird transcriptome assembly whereas 642,206,263 reads of twelve Blackbirds were created for the Blackbird blood transcriptome, but in this dataset PE and SE reads were included (Franchini et al., 2017). Concerning the transcript length, the Blackbird seems to have a longer transcriptome with 156,342,963 bp than the Blue Tit with 58,500,000 bp, including only exons (Mueller et al., 2016).

Our assembled transcriptome was annotated using the unfiltered protein database. The number of hits was still very high after filtering the results for only well characterized hits (no hits including the words unknown, predicted or hypothetical). It should be considered that using the separation of well and poorly characterized hits may lead to missing out possible avian specific transcripts due to the lack of information that currently exists. However, taking an unfiltered or less restrictive filtered approach into account, can give additional information to get a better impression of the available sequences in the assembled transcriptome. Less restrictive filtered approaches could, for example, filter for vertebrates, avian species, songbirds or a combination of specific species. For example, Laine et al. (2016) used a combination of the databases of chicken, Zebra Finch, Collared Flycatcher (*Ficedula albicollis*), and Ground Tit in a genomic study targeting the Great Tit.

Additionally, it also should be considered that in this approach the RNA was pooled from several tissues. By pooling the tissues, we may lack the ability to distinguish between tissue-specific isoforms. Furthermore, different true transcripts could have been merged together so that the probability to find matches of high hit identity could have been reduced.

All genes, which occur in the brain, heart, and liver are also present in the pool due to the inclusion of the single tissues into the pool (Fig. 2). This proves accuracy in sequencing techniques and bioinformatic analyses applied. In addition, more genes were found in the pool, because the pool combined 14 tissues. The most expressed gene in the pool are not described proteins until now, thus, there are a lot of proteins in the Common Blackbird, for which we cannot even assume the function (Table 2). An uncharacterized protein is the second most expressed gene and serum albumin is the third one, which is found in the blood and is a carrier protein. Synaptosomal-associated protein 25 is the most expressed gene unique to the brain and belongs to the trans-SNARE complex, which brings the synaptic vesicle and the plasma membranes together. The most expressed gene unique to the heart is the myosin regulatory light chain 2A, cardiac muscle isoform, which plays a role in the development and function of the heart. In the liver, the most expressed unique gene is the fibrinogen alpha chain, which is cleaved by thrombin to fibrin, the most abundant component of blood clots.

SNPs are used in several kinds of studies especially concerning population genetics and phylogeography as reviewed by Kraus & Wink (2015). Furthermore, Huang, Huang & Cheng (2013), for example, surveyed the association of SNPs within the ovalbumin gene with duck hatchability. In this study, within the sequences of the *de novo* assembled transcriptome, numerous SNPs were found. Not all of the identified genes carrying SNPs will be suitable for the selection of candidate or marker genes and those that will still have to be validated. However, to our knowledge, only a few SNPs have been identified so far and only the dopamine receptor D4 (DRD4) gene, exon 3 with a length of 561 bp matched in our draft transcriptome (Mueller et al., 2013). Nevertheless, in the sequence of exon 3 in the dopamine receptor D4 gene, five SNPs were detected (Mueller et al., 2013), which were not found in the transcript of this study. In comparison to our study, we found a longer transcript sequence of more than 894 bp long and in this sequence only four SNPs were located. The other genes from Mueller et al. (2013) could not be found in our dataset. By providing the identified SNPs with the respective nucleotide sequences, this study gives sufficient resources for future genetic studies concerning SNP approaches in protein coding genes using the Common Blackbird as study organism.

## Conclusions

NGS technologies and bioinformatics software have already proven to be potent and important tools for many scientific disciplines, especially in (molecular) ecology. By using those tools, we made a first step to the investigation of transcriptomics of an ecologically already well-studied bird species, the Common Blackbird. We established a *de novo* assembled transcriptome that will be usable for several downstream analyses, e.g., differential gene expression, SNP identification as well as the raw sequences retrieved from the NGS for re-assembly or other approaches. Therefore, new high throughput methods for sequencing and functionally describing proteins are needed so that the expansion of protein information can keep up with the growing amount of genomic and transcriptomic information. An increased knowledge of the proteins that the already known genetic sequences encode for would improve annotations of other, non-model, birds and, therefore, would improve downstream analyses like differential gene expression. Future studies should validate the provided sequences by using qPCR, for instance. Furthermore, these results will help addressing questions of urban ecology and evolutionary biology that, for example, concern the role of gene expression in phenotypic differences between rural and urban individuals and, thus, in the ability to successfully colonize urban habitats by a statistically relevant number of Blackbirds. Therefore, more individual tissues should be investigated to link a specific gene expression pattern to the correct function.

##  Supplemental Information

10.7717/peerj.4045/supp-1 Table S1 Pool contigs with annotation

10.7717/peerj.4045/supp-2 Table S2 Brain contigs with annotation

10.7717/peerj.4045/supp-3 Table S3 Heart contigs with annotation

10.7717/peerj.4045/supp-4 Table S4 Liver contigs with annotation

10.7717/peerj.4045/supp-5 Table S5 Further information about identified SNPs The table is provided as created by BCFtools.

## Additional Information and Declarations

### Field Study Permissions

The following information was supplied relating to field study approvals (i.e., approving body and any reference numbers): Kreisforstamt, Landratsamt Rhein-Neckar-Kreis approved this study.

### Data Availability

The following information was supplied regarding data availability: STUDY: PRJNA418314 (SRP124961) SAMPLE: a5 (SRS2685506) EXPERIMENT: a5ra (SRX3391025) RUN: a5rar.fq.gz (SRR6289864) EXPERIMENT: a5rb (SRX3391026) RUN: a5rb.fq.gz (SRR6289863) EXPERIMENT: a5rh (SRX3391027) RUN: a5rh.fq.gz (SRR6289862) EXPERIMENT: a5rl (SRX3391028) RUN: a5rl.fq.gz (SRR6289861) SAMPLE: a4 (SRS2685507) EXPERIMENT: a4ra (SRX3391029) RUN: a4raf.fq.gz (SRR6289860) EXPERIMENT: a4rb (SRX3391030) RUN: a4rb.fq.gz (SRR6289859) EXPERIMENT: a4rh (SRX3391031) RUN: a4rh.fq.gz (SRR6289858) EXPERIMENT: a4rl (SRX3391032) RUN: a4rl.fq.gz (SRR6289857).
